# Parsonage-Turner Syndrome Following SARS-CoV-2 Infection: A Systematic Review

**DOI:** 10.3390/biomedicines11030837

**Published:** 2023-03-09

**Authors:** Amalia Cornea, Irina Lata, Mihaela Simu, Elena Cecilia Rosca

**Affiliations:** 1 Department of Neurology, Victor Babes University of Medicine and Pharmacy Timisoara, Eftimie Murgu Sq. no. 2, 300041 Timisoara, Romania; 2 Department of Neurology, Clinical Emergency County Hospital Timisoara, Bd. Iosif Bulbuca no. 10, 300736 Timisoara, Romania

**Keywords:** Parsonage-Turner syndrome, neuralgic amyotrophy, SARS-CoV-2, COVID-19, systematic review

## Abstract

Parsonage-Turner syndrome (PTS) is an inflammatory disorder of the brachial plexus. Hypothesized underlying causes focus on immune-mediated processes, as more than half of patients present some antecedent event or possible predisposing condition, such as infection, vaccination, exercise, or surgery. Recently, PTS was reported following the severe acute respiratory syndrome coronavirus 2 (SARS-CoV-2) infection. We aimed to investigate data on PTS triggered by SARS-CoV-2 infection to provide an extensive perspective on this pathology and to reveal what other, more specific, research questions can be further addressed. In addition, we aimed to highlight research gaps requiring further attention. We systematically reviewed two databases (LitCOVID and the World Health Organization database on COVID-19) to January 2023. We found 26 cases of PTS in patients with previous SARS-CoV-2 infection. The clinical and paraclinical spectrum was heterogeneous, ranging from classical PTS to pure sensory neuropathy, extended neuropathy, spinal accessory nerve involvement, and diaphragmatic palsy. Also, two familial cases were reported. Among them, 93.8% of patients had severe pain, 80.8% were reported to present a motor deficit, and 53.8% of patients presented muscle wasting. Paresthesia was noted in 46.2% of PTS individuals and a sensory loss was reported in 34.6% of patients. The present systematic review highlights the necessity of having a high index of suspicion of PTS in patients with previous SARS-CoV-2 infection, as the clinical manifestations can be variable. Also, there is a need for a standardized approach to investigation and reporting on PTS. Future studies should aim for a comprehensive assessment of patients. Factors including the baseline characteristics of the patients, evolution, and treatments should be consistently assessed across studies. In addition, a thorough differential diagnosis should be employed.

## 1. Introduction

Neuralgic amyotrophy, or Parsonage-Turner syndrome (PTS), is a peripheral nervous system disorder with two prominent features: severe pain and significant muscle atrophy. The symptoms primarily affect the forequarter region of the body, including the cranial, shoulder, upper limb, and ipsilateral chest wall. A precipitating antecedent event, or a trigger, can be recognized in most cases.

In the mid-1800, two separate disorders, serratus magnus paralysis and post-infectious paralysis, were initially described, indicating the muscle involved (serratus anterior) and that the syndrome followed an infection. Subsequently, two other entities were reported: serogenic neuropathy and vaccinogenic neuropathy, relating to their presumed triggers. Later, several other entities were identified and labeled with terms relevant to their location, pathology, or trigger. In 1948, Parsonage and Turner recognized the common characteristics of these conditions, concluding that they represented a single entity with various presentations [1]. They coined the term neuralgic amyotrophy based on recognizing these two major clinical features: severe pain and significant muscle wasting. Ultimately, a unifying clinical triad was identified: an antecedent event or trigger, sudden onset of intense forequarter region pain, and severe weakness and wasting of regional muscles. Nowadays, it is widely recognized that these disorders represent phenotypic variations of a single syndrome.

### 1.1. Epidemiology

Traditionally, PTS has been considered a rare disorder, with an annual incidence estimated at 1.64 cases per 100,000 population [2]. Nonetheless, the actual incidence is much higher, as the condition is underrecognized. A prospective study reported an incidence of 1 case per 1000 population [3]. PTS presents two major forms: sporadic and hereditary. The sporadic form is more frequent, affecting primarily young to middle-aged adults, with a mean age of onset of 40 years; the male-to-female ratio has been reported to be 2–2.3 [4]. The hereditary form has a mean age of onset of 25 years [4].

### 1.2. Clinical Presentation

Van Alfen and van Engelen presented one of the most comprehensive case series of PTS to date. They reported on 246 patients in a tertiary care setting [3], defining the typical clinical characteristics of PTS. In this series, PTS manifested with the primary onset of severe neuropathic pain, followed by patchy upper limb paresis, ranging from isolated anterior interosseous nerve palsy to severe bilateral paresis of both upper limbs. Nonetheless, despite significant variations in presentation, the triad comprising of (i) a recognized trigger, (ii) forequarter region pain, and (iii) forequarter region muscle weakness and wasting are distinctive, enabling easy diagnosis. Even if the triad is incomplete, PTS is typically identified based on the two most significant clinical features (severe pain, muscle weakness, and/or atrophy) that are almost invariably present. Although most patients report focal pain as the primary chief complaint, a focal sensory loss is rarely found; when present, it is usually minor. Accordingly, the neurologic examination abnormalities primarily involve the motor system [5].

The pain typically has a sudden onset; usually, it awakens the patient from sleep or is noted immediately upon awakening. The pain increases in intensity over several hours. Due to its severity, it leads the patient to seek medical attention promptly. The pain is exacerbated by movements of the shoulder or the upper extremity. Notably, it is not aggravated by head or neck movement, differentiating it from acute radiculopathies. After 1–2 weeks, the symptom resolves or is replaced by dull aching pain. Despite severe pain, cutaneous sensory axon involvement producing numbness is rare [6].

Forequarter motor deficit and muscle wasting follow the pain. The symptoms are generally identified when the pain subsides and the patient starts to use the affected limb. The weakness is sometimes not initially noted; muscle atrophy is the identified feature. Muscle wasting usually appears within a few weeks of the PTS onset. Rarely, weakness and atrophy might be absent.

### 1.3. Etiology and Pathophysiology

Triggering events are reported to be associated with at least 50% of PTS cases. The most common is an upper respiratory infection or influenza-like illness. Bacterial infections that may trigger PTS include pneumonia, malaria, typhus, diphtheria, rheumatic fever, borreliosis, dysentery, sepsis, rickettsia coroni, and bartonella henselae (cat claw). Among viruses, influenza, cytomegalovirus, herpes virus, varicella-zoster virus, parvovirus B19, Epstein-Barr, coxsackie, Echo 13/30 virus, smallpox, poliomyelitis, and hepatitis B were reported to trigger PTS [7]. Furthermore, approximately 10% of PTS patients were found to have a concomitant hepatitis E virus infection in the acute phase, thereby explaining previous reports of elevated liver enzymes in some [3]. Other triggers include immunizations and vaccinations, medical or surgical procedures, childbirth, unaccustomed physical activity, and trauma, including the minor trauma associated with falling (without apparent injury) and intravenous procedures (intravenous therapy, intravenous contrast, or intravenous blood withdrawal) [7]. In addition, PTS has been reported after administering some medications, including nivolumab [8] and botulinum toxin [9].

These triggers are considered to activate the immune system in susceptible individuals. The latency between the trigger and PTS is generally reported to range from several hours up to 4 to 6 weeks. Nonetheless, in about two-thirds of patients, the pain starts during the first week [6].

Most triggering factors indicate an underlying autoimmune process with selected peripheral nerve inflammation. Furthermore, pathological studies from nerve biopsies in acute PTS report the presence of lymphocytic inflammatory infiltrates in the affected nerves. The initial inflammation causes intraneural edema. The swollen fascicles are less flexible, and the motion of a nearby articulation induces kinking. Repetitive kinking and rotation of the nerves can lead to constriction and fascicular entwinement. Many patients report intense physical activity of the upper body before the onset of PTS [3]; this indicates that mechanical stress to the nerves plays a predisposing role [3]. Repeated microtrauma to the brachial plexus nerves of the plexus might determine an increase in the permeability of the blood-nerve barrier, opening the endoneurial space to immune factors and enabling the autoimmune process [10].

The sudden onset, monophasic course of PTS, association with preceding infection, serum sickness, vaccinations, and use of immunomodulating agents all support immune-mediated pathology. In addition, this hypothesis is supported by the involvement of both immune mechanisms, humoral and cellular, the existence of focal chronic inflammatory infiltrates, edema, and onion bulb appearance. The endoneurial and epineurial vessels are surrounded by mononuclear inflammatory infiltrates without features of necrotizing vasculitis. Furthermore, the PTS patients were reported to present altered lymphocyte subsets (decreased CD3 levels and increased CD4/CD8 ratios due to decreased CD8 levels), antiganglioside and anti-peripheral nerve myelin antibodies, and terminal complement activation products [11,12,13,14]. Oligoclonal bands were reported in the cerebrospinal fluid (CSF) [11,12,14]. Moreover, triggers of PTS, such as an upper respiratory infection, also represent triggers for other autoimmune diseases, including acute and chronic inflammatory demyelinating polyradiculoneuropathy [6].

The hereditary form of PTS has been described in approximately 200 families worldwide [15]. This form accounts for about 10–19% of PTS cases [4]. The patients present similar clinical features with the sporadic form, including antecedent triggers, intense pain, muscle weakness, and atrophy. Differences between the hereditary and sporadic forms include the age of onset, the frequency of recurrences, and some morphological features [6]. For example, although hereditary and sporadic forms are generally present in the third and fourth decades, and both forms may present in the first decade, children are more frequently affected by the hereditary form.

The hereditary form of PTS transmits in an autosomal dominant manner. Researchers found that approximately 55% of gene mutations in North American families affect the SEPT9 gene on chromosome 17 q and show high penetrance of 80–90% [10,16,17]. The genetic abnormality in the other 45% of these families is unknown, suggesting that hereditary PTS is a genetically heterogeneous syndrome.

Electrodiagnostic research indicates that the primary pathophysiology associated with both forms of PTS comprises axon disruption with Wallerian degeneration. Following Wallerian degeneration, conduction failure occurs as action potentials can no longer propagate along the axon. In contrast to focal myelin disruption, which remains focal, focal axon disruption presents with distant effects. Clinically, early muscle wasting and long recovery periods are also consistent with these findings. However, rarely (less than 1% of cases), PTS patients with sporadic forms might present focal demyelination [6].

### 1.4. Diagnostic Workup

Laboratory investigations have only a limited diagnostic value. They may aid in identifying specific infections associated with the onset of PTS and are used primarily for some differential diagnoses [10].

Electrodiagnostic investigations are widely used, supporting the diagnosis of PTS. Needle electromyography (EMG) is an invasive but valuable tool to detect muscle denervation. However, denervation may take up to four weeks to be fully apparent in EMG, and early measurements can thus be of limited value [18]. Nerve conduction studies (NCS) indicate the site of the lesion [5]. Nonetheless, in the subacute stages, after some reinnervation has appeared, the parameters of affected nerves may be normal, limiting the sensitivity of this diagnostic tool [10].

Furthermore, conduction slowing or blockage may be technically challenging to detect in some specific nerves due to their anatomical location. Sensory abnormalities are absent on NCS in 80% of clinically affected nerves [19]. Therefore, a normal NCS does not exclude a PTS diagnosis.

There is no differential diagnosis when a patient presents a PTS with paralysis of a long thoracic nerve and a left suprascapular nerve simultaneously. Otherwise, the main differential diagnoses include cervical root and shoulder joint disorders [9]. In patients with mononeuropathies, an entrapment neuropathy associated with a fibrous arcade, synovial cyst, or lipoma may cause the symptoms. Depending on clinical and additional investigations, meningoradiculitis, neoplastic plexopathy, or vasculitis could be considered. If the patient presents no pain, the differential diagnosis includes chronic idiopathic demyelinating polyneuropathy, multifocal motor neuropathy, Lewis Sumner syndrome, hereditary neuropathy with liability to pressure palsy, and facio-scapulo-humeral myopathy [7].

Recently, PTS was reported following the severe acute respiratory syndrome coronavirus 2 (SARS-CoV-2) infection. Our objective is to provide a comprehensive report on existing literature by investigating data on PTS triggered by SARS-CoV-2 infection, provide an extensive perspective on this pathology, and reveal what other, more specific, research questions can be further addressed. In addition, we aim to highlight research gaps requiring further attention. Therefore, we aim to evaluate the clinical, laboratory, neurophysiological, and neuroimaging features of PTS in patients with Coronavirus disease 2019 (COVID-19) and explore possible links in this pathology.

## 2. Materials and Methods

This systematic review was performed following the guidelines of the Preferred Reporting Items for Systematic reviews and Meta-Analyses extension for Scoping Reviews (PRISMA-ScR) [20,21,22,23] (see Supplemental Materials S1) and the current recommendations on the synthesis of case reports and case series [24].

The research questions were defined based on the Population, Concept, and Context (PCC) of the review, as recommended by the Joanna Briggs Institute [20]:

- Is there a relationship between SARS-CoV-2 infections and the apparition of PTS?
- If yes, what are the clinical features?
- What do we know about laboratory, neurophysiological, and neuroimaging investigations?
- Which are the presumptive mechanisms underlying PTS?
- What interventions might work?
- What do we know about the evolution of PTS after SARS-CoV-2 infection?

In order to identify the extent of the current research on PTS after SARS-CoV-2 infection, we searched LitCOVID, the World Health Organization database on COVID-19 (to 17 January 2023), using the following search strings “Parsonage AND Turner”, and “brachial”. As these databases are curated for SARS-CoV-2 infection articles, we did not need to use search terms like “coronavirus”, “COVID-19”, or “SARS-CoV-2”. We screened for additional studies using the reference lists of relevant research papers. As we aimed to generate an extensive list of research suitable for answering our questions, we did not apply any search filters and language restrictions.

Two authors reviewed the title, abstract, and full text (when needed) of all retrieved articles, assessing whether the study met the inclusion criteria. A third reviewer’s opinion was considered if disagreements were not solved through discussion.

The PCC mnemonics for this systematic review were children and adults (over 18 years old) (P), with studies investigating patients with PTS (C) in the context of previous or concurrent SARS-CoV-2 infection (C). We included case reports, case series, and prospective or retrospective observational and interventional studies. Conference abstracts were also included if the authors did not publish a full paper on the study.

We excluded commentaries, opinions, and narrative reviews but examined their reference lists for possible inclusions. Also, we excluded patients with COVID-19 reported to present neuropathy after prone positioning.

We extracted data to a pro forma template piloted on five randomly selected articles and adjusted the template as necessary. One reviewer extracted all relevant information, and a second reviewer checked the data.

Our primary scope was to provide an overview of the evidence reported on PTS triggered by SARS-CoV-2 infection, regardless of the risk of bias in the included studies [20]. Therefore, we did not perform a formal evaluation of the methodological quality of the included studies.

The protocol was not registered to any database.

## 3. Results

**Figure 1:**
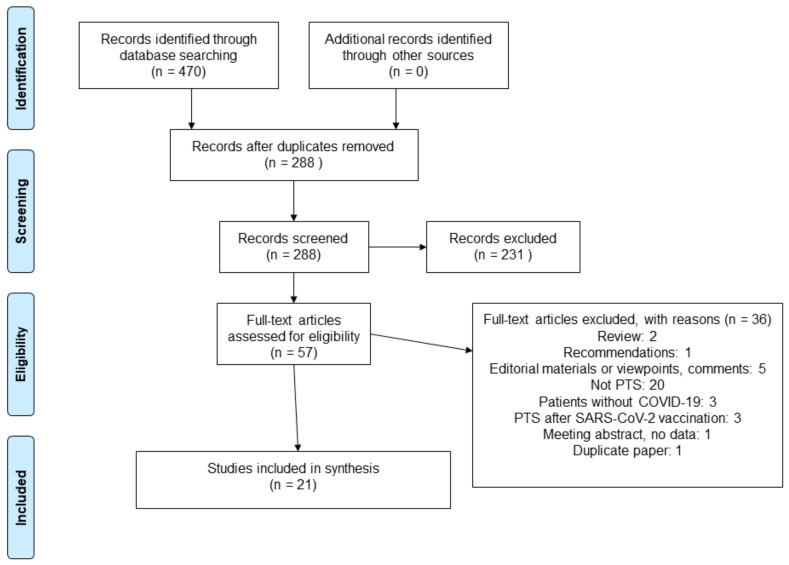
Flow chart showing the process for inclusion of studies. Legend: PTS: Parsonage-Turner syndrome. SARS-CoV-2: severe acute respiratory syndrome coronavirus 2.

The literature search resulted in 470 records. After deduplication, 288 articles were included. Finally, we identified 57 papers on PTS in patients with COVID-19 to assess in full text; 21 papers were ultimately included [25,26,27,28,29,30,31,32,33,34,35,36,37,38,39,40,41,42,43,44,45]. The screening and selection of papers were conducted by one reviewer and cross-checked by a second author. Disagreements were managed by discussing between the two screeners or having a third author arbitrate. The PRISMA diagram with the selection process is illustrated in Figure 1.

This systematic review included 21 articles reporting on 26 cases of patients from the USA (8 cases) [25,27,32,38,40,44], France (6 patients) [34,36,41,43], Italy (3 cases) [29,31,45], Spain (1 case) [26], Germany (1 case) [42], Hong Kong (1 case) [33], Singapore [39], Kuwait (1 case) [37], Iran (1 case) [28], Chile [35], and Mexico (2 cases) [30].

The patients were aged between 17 and 76 years, most of them being males (21/26, 80.8%). The publication dates ranged from 2020 to 2022. The detailed study characteristics are presented in Supplemental Materials S2.

### 3.1. Medical History

Regarding the immunological status of the patients, one report notes that the patient was vaccinated a few months prior to PTS [41], but the authors provide no further information on the vaccine; another patient was vaccinated with Moderna mRNA 1273 four months prior to PTS [39], and two patients had no recent vaccination [31,35]. One case received kidney transplantation due to polycystic kidney disease and was under treatment with prednisolone, tacrolimus, and mycophenolate mofetil [28]. Two patients, a sister and a brother, had a history of steroid allergy [30]. Two patients were investigated for autoimmune diseases, but the vaccination status is not reported [34]. For the remaining 17/26 (65.4%) PTS cases, the authors did not provide information on vaccination status or other immunosuppressing states.

Nine (34.6%) patients presented significant comorbidities: clavicle fracture [25], familial history of PTS [30], history of work-related shoulder pain [32], shoulder arthroplasty [32], psoriatic arthropathy in remission [33], an episode of anterior dislocation of the shoulder [41], or prior rotator cuff repair performed 2.5 years previously [44].

### 3.2. Clinical Picture

The PTS had an acute onset in all cases (26/26, 100%). In two patients (2/26, 7.7%), the onset time of PTS was unclear [26,29]. The neurological complaints were reported to occur simultaneously with COVID-19 in 5/24 (20.8%) patients [30,32,41,45], and within one week of infection in 3/24 (12.5%) cases [25,37,39]. Nine individuals (9/24, 37.5%) developed PTS after more than seven days but within one month of infection [28,31,32,33,35,36,42,43]. In 7/24 (29.2%) patients, PTS symptom onset occurred after more than a month from COVID-19 infection [27,32,34,38,40,44]. In two patients, the time of onset of PTS was unclear.

Pain was present in most cases (25/26, 96.2%). Only one patient (1/26, 3.8%), hospitalized in the intensive care unit (ICU) with Acute Respiratory Distress Syndrome (ARDS), had neuralgic amyotrophy affecting C5–C6 nerve roots, the lateral pectoral and phrenic nerves; he presented hypoesthesia, motor deficit, muscle wasting, and diaphragmatic palsy [43]. The characteristics of pain in each case are presented in Table 1.

A motor deficit was present in 21/26 (80.8%) patients [26,27,28,29,32,33,34,35,36,38,39,40,41,42,43,44,46]. Muscle strength was normal in 3/26 (11.5%) patients; two presented a pure sensory PTS [31,45], and one case is unclear: the authors report a normal motor function, but eight weeks later, he had scapula winging [25]. In addition, in two familial cases (2/26), the authors do not mention any motor deficit, but the patients presented deltoid, supraspinatus, and scapular muscle wasting [30].

Muscle wasting was reported in 14/26 (53.8%) patients [25,26,27,29,30,32,34,35,37,41].

Paresthesia was noted in 12/26 (46.2%) PTS individuals [25,26,27,30,32,33,37,39,40,42]. However, a sensory loss was reported in 9/26 (34.6%) patients [26,28,31,33,37,42,43,44,45].

The clinical picture of individual cases is presented in Table 2. The extended data can be found in Supplemental Materials S1.

### 3.3. Ancillary Investigations

Among 26 patients, NCS was performed in 11 cases (11/26, 42.3%) [27,31,33,34,35,36,37,42,45], and EMG in 18 cases (18/26, 69.2%) [25,28,31,32,33,34,35,36,37,41,42,43,44,45]. The NCS findings varied depending on the nerve fibers affected and the timing of the investigation. The authors reported acute motor axon loss signs [26], normal latencies with an important reduction of compound action potentials (CMAP) amplitude [34], signs of subacute plexopathy [35], and normal findings three months after PTS onset [33]. In addition, a few patients also presented an absence of sensory responses [26] or reduced sensory nerve activation potential amplitude [31]. Some authors noted that they performed electrophysiological studies for four patients [26,29,30]. Likewise, the EMG results were also heterogeneous: signs of denervation [25,29,32,34,36], patchy plexopathy [40], positive sharp waves and fibrillation [35], chronic trunk plexopathy with reinnervation [27], and normal findings five weeks after PTS symptoms onset [31].

A neuromuscular ultrasound investigation was employed in 5/26 (19.2%) cases [25,29,31,42,43]. The findings included enlargement of the affected nerves [25,29,42], multifocal damage of the nerves [29], and amyotrophy [43]. Also, normal findings were reported in a patient with pure sensory PTS [31].

Diaphragmatic ultrasound was useful for assessing diaphragmatic dysfunction [43].

MRI was performed in 17/26 (65.4%) cases. The most common finding was muscle edema (10/17, 58.8% cases) [26,27,29,37,38,41,42,43,44], followed by hyperintensity of the affected fascicles (8/17, 47.1% cases) [29,33,36,39,40,43,45], and muscle atrophy (5/17, 29.4% patients) [34,41,43,44]. Contrast enhancement of the affected nerve was reported in one patient [42]. Also, on MRI diffusion neurography of the brachial plexus, the measurements of anisotropic fractions and apparent diffusion coefficient (ADC), compared with the normal side, identified a tendency to isotropy, with nervous elements structural disorganization, consistent with PTS. With contrast medium, no reinforcement foci were noted. The authors suggest this signal asymmetry was probably due to inflammation [35]. Other authors note a short inversion time inversion recovery (STIR) hypersignal on the path of the affected nerve bundles, without caliber abnormality or lesion detectable by MRI [43] or segmental diffusion-weighted imaging (DWI) restriction and corresponding apparent diffusion coefficient (ADC) low signal of the nerve trunk [45]. Also, hourglass constrictions were detected in one case [29].

A nerve biopsy from a medial brachial cutaneous nerve was performed in one case, demonstrating marked axonal loss [40].

### 3.4. Treatment

Non-steroidal anti-inflammatory drugs (NSAIDs) were prescribed initially for 8/26 (30.8%) cases, with minimal or no effect [25,26,27,30,33,44]. Two patients (2/26, 7.7%) also received muscle relaxers [25,44]. Other authors report an initial administration of acetaminophen (2/26, 7% of patients) with minimal relief [31,37].

As the patients presented with severe pain, 5/26 (19.2%) received gabapentin or gabapentin-type neuromodulators [25,30,33,45]. Pregabalin was tried for 3/26 (11.5%) cases [33,35,37], and duloxetine was given to one patient [45]. Five patients (5/26, 19.2%) were treated with opioids [26,30,37,44].

In addition, one patient also received local injections with steroids and lidocaine, leading to a slight improvement [37]. Two patients received pain medication, but the authors did not specify precisely the type of drugs (2/26, 7.7%) [34].

Corticosteroids were administered in 12/26 (46.2%) PTS cases [26,28,29,32,34,36,37,38,41,42,45]. One patient received intravenous methylprednisolone (1000 mg daily for 5 days), but it was stopped due to dermatological side effects. A course of intravenous immunoglobulins (IVIG) (25 g/day for 5 days) was also employed, with partial relief of pain and no improvement in muscle power [37]. The rest of the patients received oral steroids. Among the patients that received steroid therapy, the authors reported the outcome in eight cases. One patient with isolated musculocutaneous involvement fully recovered at one month [29]. Another PTS patient that received oral prednisolone (25 mg) for three weeks, followed by tapering, presented partial improvement of muscle strength at 21 days after the initiation of the treatment. At the 2-month follow-up, the patient’s shoulder examination was normal, without pain or functional limitations [28]. Four patients demonstrated partial improvement on follow-up visits at different time intervals (ranging from two months to six months) [32,36,41,45]. In one individual, the neurological examination remained the same at eight weeks [37]. Another case reported pain relief at 7- and 14-days, but the motor and sensory examinations remained the same [42].

In two patients, a brother and sister with a history of steroid allergy, the authors administered extended-release pirfenidone, starting on day 22, considering its potential anti-inflammatory action [30]. On day 26 post-infection, the symptoms of neuralgia subsided [30].

Rehabilitation was recommended for 11/26 (42.3%) PTS patients [26,32,33,34,35,41,43,44].

One patient with pure motor PTS received no treatment (1/26, 3.8%). Nonetheless, the evolution was favorable, with the complete disappearance of the symptoms after three months [36]. The authors provided no information on the treatment [39,40].

### 3.5. Evolution

In 7/26 (26.9%) patients, the authors did not report on the evolution of PTS [26,32,34,38,40,43]. Among the 19 patients with reported outcomes, 5/19 (26.3%) had a complete remission of symptoms at 1 month [29], 2 months [28], 3 months [27,36], and 6 months follow-up visits [35], respectively.

The clinical examination was found to be improved in 11/19 (57.9%) patients. Nonetheless, the timing of the follow-up visits was heterogeneous: 13 days [39], approximately 3 weeks [30], 6 weeks [31], 2 months [25,45], 3 months [33], 4 months [32,36], 6 months [41], 8 months [44], and 9 months [32].

### 3.6. SARS-CoV-2 Infection

The COVID-19 diagnosis was based on a positive real-time reverse-transcription polymerase chain reaction (RT-PCR) test in 15/26 (73.1%) patients [25,27,28,30,31,34,36,37,41,42,43,45]. However, the cycle threshold (Ct) of the positive RT-PCR test was not specified in any cases. In 9/26 (34.6%) patients, the authors did not specify how the COVID-19 diagnosis was obtained [26,29,32,33,39,40,44]. Two patients (2/26, 7.7%) were not tested by RT-PCR and the diagnosis of SARS-CoV-2 infection was made retrospectively. One patient presented anti-SARS-CoV-2 antibodies suggesting prior infection/exposure (elevated IgG, normal IgM) [35]. Another case had a negative RT-PCR but positive IgG antibodies a few weeks after the respiratory illness [38].

Regarding the COVID-19 severity, 4/26 (15.4%) patients had mild disease [33,42,44,45], one patient presented moderate to severe infection (1/26, 3.8%), and 9/26 (34.6%) had severe illness [25,26,27,29,32,34,40,43]. The severity of the SARS-CoV-2 infection was not reported for 12/26 (46.2%) of cases [30,31,32,35,36,37,38,39,41]. Among the patients with severe COVID-19, eight (8/26, 30.8%) required ICU treatment [26,27,29,32,34,40,43]. Four patients (4/8, 50%) developed PTS symptoms several days or weeks after ICU discharge or extubation [26,27,32,34], including one with extended PTS [27]. The data on ICU stay and PTS symptoms is presented in Table 3.

## 4. Discussion

The present systematic review identified 26 cases of PTS following SARS-CoV-2 infection. The study population included 80.8% males, a more significant proportion than commonly reported in the literature in PTS. For example, the cohort evaluated by van Alfen et al. consisted of 67.5% males [4]. However, as PTS is considered to present an autoimmune origin, one would expect to present a female predominance. Therefore, an unknown sex-specific factor in this pathology may make male patients more prone to attacks [4]. In addition, a family history was present in two cases [30], but no genetic testing was performed. This proportion (7.7%) is lower than reported in the general population (19%), but several authors in our review did not report on the family history of their patients.

In our series, 15/26 (57.7%) patients experienced severe pain. However, the pain intensity was not clearly presented in 10/26 (38.5%) cases. One patient did not present pain [43]. Therefore, among 16 patients with information on pain intensity, 93.8% had severe pain. This data is similar to other reports in the literature [4,6]. Usually, the pain in PTS emerges within a few hours and, in most cases, the attacks begin at night [4,15]. However, most articles in our review did not provide detailed patient pain data. Among 25 patients with pain, 22 (88%) had unilateral symptoms and three (12%) presented bilateral symptoms in an asymmetric pattern [26,37,38]. Our percentage of patients with unilateral pain is higher than the data found by other authors in patients with PTS. For example, van Alfen reported that, in 71.5% of patients, the pain was unilateral, and in 28.5%, it was bilateral [4]. In 5/25 (20%) patients with pain, the symptom caused sleep disturbances [26,32,33,44]. In our systematic review, the percentage is much lower than reported in the literature (93.5%) [4], possibly because the authors do not mention anything about sleep quality in 20/25 (80%) cases. An increased mechanical sensitivity (pain elicited by movement, pressure, or touch of the affected limb) was reported in 5/25 (20%) of patients (4 males and 1 female) [32,33,37,38,42]. In one case (1/25, 4%), the pain was not alleviated or aggravated by shoulder movement [44]. Nonetheless, these aspects are not presented in most cases (19/25, 76%).

In PTS, many patients describe three pain phases during the attack. The constant initial pain may be followed by intense neuropathic stabbing or shooting pains, often elicited by motion, lying on, or prolonged limb posturing. About two-thirds of PTS cases reported further subsequent persisting musculoskeletal pain. This later pain type is usually localized to the origin or insertion of the paretic or compensating muscles, primarily in the periscapular, cervical, and occipital regions [4]. However, such a detailed description of the patient’s symptoms was unavailable in the studies included in this review.

The timing of other symptoms and signs that follow the pain was reported in 8/25 (32%) patients [25,32,35,36,37,38,42,44]. Among them, 6/8 (75%) presented additional symptoms within 2 weeks; in 2/8 (25%) patients, the motor deficit was reported after a longer time interval (seven weeks [35] or a few weeks [32]). Our findings are similar to other reports, where authors found that 27.2% of all cases of paresis did not manifest themselves until >2 weeks after the onset of pain [4]. A recent study with an in-depth analysis of PTS patients found that, in about one-third of patients, there is an increment of the motor deficit over days (8.6%), weeks (16%), or months (5.6%) [4]. However, we did not find reports on the aggravation of the motor deficit.

Among the 24 patients with a motor deficit or muscle wasting, in ten patients, the motor deficit was assessed using the Medical Research Council (MRC) grading system (see Supplemental Materials S2). The intensity varied from 1/5 to 4/5, with 7/10 (70%) of individuals presenting a maximum deficit of 3/5 or 4/5 [27,29,32,36,39,41] and 3/10 (30%) cases presenting severe paralysis with an MRC of 1/5 or 2/5 [32,35,37]. This contrasts with the literature, where about two-thirds of patients presented severe motor deficits [4]. In our review, among individuals with reported outcomes, 26.3% had a complete recovery.

In addition, the clinical assessment revealed that 53.8% of patients presented muscle atrophy, while data from the literature indicate more significant proportions, ranging from 75.4 to 88.5% [4]. This discrepancy could be due to the timing of examination, as the median time for atrophy to first appear was reported to be 5 weeks [4], or to the fact that in our series, the motor deficit was not severe in most cases. Furthermore, muscle weakness and wasting might go unnoticed. First, weakness is only sometimes appreciable in the early PTS stage if pain limits the patient’s movements. Also, synergistic muscles may mask the motor deficit, or an overlying muscle may mask the muscle wasting (e.g., the trapezius muscle masks supraspinatus muscle atrophy, the biceps muscle may mask brachialis muscle atrophy) [6].

The pattern of sensory symptoms in our series also differs from the results of other studies investigating PTS. Paresthesia was noted in 12/26 (46.2%) PTS individuals [25,26,27,30,32,33,37,39,40,42]. However, a sensory loss was reported in 9/26 (34.6%) patients [26,28,31,33,37,42,43,44,45]. The sensory symptoms and signs were present alone or in combination with motor findings in different patterns. Although previous research demonstrated that in more than half of PTS patients, there is no recovery on follow-up [4], the evolution of sensory symptoms was poorly reported in PTS patients following SARS-CoV-2 infection, with the authors focusing on motor recovery.

In addition, in our review, no patient presented autonomic nervous system involvement (e.g., vegetative and trophic skin changes, edema, temperature dysregulation), although they have been reported in 15.4% of PTS patients [4]. Involvement of nerves outside the brachial plexus was reported in several patients, including the lumbosacral plexus [27], phrenic nerve [43], and spinal accessory nerve [34].

The lumbosacral nerves may be rarely affected in hereditary PTS forms [6], but authors investigating large series of sporadic series of PTS did not report any lower extremity muscle involvement [5]. Furthermore, when the lower extremity muscle involvement is not concomitant with the episodes of forequarter region weakness, it is uncertain that they represent the same disorder [6]. For example, lumbosacral radiculoplexus neuropathy and PTS share the same clinical features: severe pain, muscle weakness, and atrophy. For the patient with extended PTS included in the present review, although he did not present genetic testing, the authors note he had no family history of neurological diseases [27].

Phrenic nerve involvement is difficult to diagnose. For example, in a study of phrenic neuropathies due to PTS, 10 of the 17 cases were isolated, with no evidence of involvement of other concomitant nerves [47]. The patients may present with unilateral or bilateral diaphragmatic palsy. When unilateral, it may be undiagnosed. In addition, when isolated, phrenic neuropathies are likely to go unrecognized if asymptomatic or if they cause only mild and transient dyspnea. They are more likely to be diagnosed when accompanied by an antecedent event or severe shoulder pain [6]. In the present review, one patient presented phrenic nerve involvement [43], but the possibility of diaphragmatic palsy might have been overlooked.

The incidence of cranial nerve involvement varies from 0% [48] to 10% [49], being more frequent in patients with hereditary forms of PTS [4]. In a study on sporadic PTS, the spinal accessory nerve was the most commonly involved cranial nerve, accounting for approximately 2% of the total lesions [5]. Two patients in our review presented spinal accessory nerve involvement [34], but they had no genetic testing, and the family history is not mentioned.

Pulmonary imaging, including chest X-ray or computed tomography results, were reported in 9/26 (34.6%) patients. These imaging methods help investigate the differential diagnosis for PTS (e.g., Pancoast tumor) and the possibility of diaphragmatic paralysis. However, in our cases, it was performed primarily for COVID-19, with rare exceptions [34,43]. Also, an MRI of the cervical spine was performed in 8/26 (30.8%) patients in order to exclude a spinal pathology [25,27,28,33,36,39,42,44]. An MRI of the shoulder was performed in 7/26 (26.9%) cases [32,34,35,38]. Although many patients presented changes on the cervical spine MRI (see Supplemental Materials S2), the results did not explain the patients’ clinical picture and course.

The most used diagnostic test was EMG (69.2% of cases), followed by MRI of the upper limb and brachial plexus (65.4% of individuals), NCS (42.3% of patients), and neuromuscular ultrasound (19.2% of cases). Only on one occasion did the authors perform a nerve biopsy (3.8% of cases). Interestingly, the MRI scan was abnormal in 16/17 (94.1%) patients. Only one case with MRI without gadolinium presented normal findings [28].

The diagnosis of PTS is primarily clinical, based on the typical history and neurologic examination findings. Nonetheless, electrodiagnostic studies are helpful. They can localize and characterize individual peripheral nervous system lesions and identify typical patterns (e.g., mononeuropathies and multiple mononeuropathies involving pure or predominantly motor nerves, severe involvement of one muscle, and spare or relative spare of others). However, a normal NCS does not exclude, with certainty, a PTS diagnosis. In addition, MRI and ultrasound studies provide information on individual lesions, potentially providing additional confirmation when required [50,51,52,53]. Although ultrasound is less valuable than MRI for brachial plexus imaging, it is much more helpful for extraplexal imaging due to its ability to follow the nerves and fascicle courses [6]. As most lesions in PTS are extraplexal, this gives ultrasonography a benefit over MRI. Other advantages of ultrasonography include better spatial resolution, lower costs, ease of side-to-side comparisons, and real-time examination [54]. Some authors prefer ultrasound imaging, considering that most PTS lesions are extraplexal [5,52] and because the MRI field of view at a given resolution restricts the detailed examination of the peripheral nervous system, with false-negative results [6].

Although there are no diagnostic blood, urine, or CSF tests for PTS, routine blood work is required to exclude emergent and treatable conditions. For example, metabolic studies may reveal increased liver enzymes, and a further hepatitis profile is warranted. Also, serology for common infections and laboratory testing for vasculitis might be required. When the patients present risk factors for specific disorders (e.g., human immunodeficiency virus infection), ancillary investigations related to these conditions are also helpful. Recent reports note that antiganglioside antibodies are present in 26% of the PTS patients tested [4]. Nonetheless, in the present review, the authors investigated the antiganglioside antibodies only in one patient [36].

During the acute phase of PTS, pain control is a priority. In general, NSAIDs and acetaminophen do not provide relief [6], and neuropathic pain medication is recommended (an antiepileptic drug, such as gabapentin or pregabalin, or a tricyclic agent, such as amitriptyline or nortriptyline) [6]. Patients with PTS following SARS-CoV-2 infection received various pain medications. Corticosteroids in different doses and regimens were administered in 46.2% of PTS cases with mixed results, from full recovery at one month to partial improvement of symptoms or no improvement. Nonetheless, randomized placebo-controlled trials are needed to evaluate the effects of corticosteroids and other medications in PTS patients.

One patient with PTS following SARS-CoV-2 infection received a course of IVIG, with partial relief of pain and no improvement in muscle power [37]. Small case series of IVIG treatment report that early treatment may shorten the disease course, being more efficient than delayed treatment [54]. Nonetheless, further research is required in this direction.

Physical therapy was recommended in 42.3% of the cases in the present review. In the acute phase, the pain is severe and may be exacerbated by limb motion. Therefore, physical therapy should be started once the pain permits movement, including range-of-motion exercises, stretching exercises, agonist muscle strengthening, and orthotic devices [6]. Surgical intervention is reserved for patients with refractory or severe disease who have failed conservative treatment. However, at least three months should be given to await any spontaneous recovery [55,56,57], but in cases where no constrictions are found on ancillary investigations, conservative treatment should be continued [58].

The data on the prognosis of PTS are variable. For example, some authors found that 36% of patients recover within one year, 75% within two years, and 89% within three years [48]. Other studies report that only 11 of 83 PTS patients had complete recovery over a 17-year follow-up [59]. Nonetheless, the likelihood of recovery for each unique lesion should be determined using the basic rules of reinnervation [6]. In patients with previous SARS-CoV-2 infection, the PTS symptoms, 26.3% had a complete remission by six months.

The limitations of the present review are primarily related to the quality of the included studies. The data extraction was challenging due to missing, incomplete, or unclear descriptions of the information. This could be due to the lack of standardized methodology and clear reporting criteria contributing to substantial methodological variation in SARS-CoV-2 studies. Furthermore, some included studies generated interesting scientific debates [60,61,62,63].

In addition, an increased possibility of bias associated with case reports and the limited inferences they provide may raise concerns. Our findings are confined by the quality and the extent of information in included reports, which were inconsistent among the 26 included patients. This concerns both the PTS and the SARS-CoV-2 infection. For example, information on previous COVID-19 is scarce in most patients. In addition, other PTS triggers, such as coinfections, intravenous procedures (e.g., intravenous therapy, contrast administration, or blood withdrawal), and certain medications, are not thoroughly assessed.

However, case reports are an appropriate study design, essential in advancing research, particularly for rare conditions. Despite the methodological constraints, observing individual patients provides important insights into a disease’s etiology, pathogenesis, natural evolution, and treatments [24]. They play a critical role in shedding light upon new events and provide first-line evidence to further test hypotheses with statistical approaches. The present systematic review highlights the necessity of having a high index of suspicion of PTS in patients with previous SARS-CoV-2 infection, as the clinical manifestations can be variable. Our findings emphasize the need for a standardized approach to investigation and reporting on PTS. Future studies should aim for a comprehensive assessment of patients. Factors such as the baseline characteristics of the patients, evolution, and treatments should be consistently assessed across studies. Also, a thorough differential diagnosis should be employed.

## 5. Conclusions

To the best of our knowledge, this is the first systematic review of PTS following SARS-CoV-2 infection. We found that, to date, only 26 cases have been reported, with various clinical and paraclinical findings. The clinical and paraclinical spectrum was heterogeneous, ranging from classical PTS to pure sensory neuropathy, extended neuropathy, spinal accessory nerve involvement, and diaphragmatic palsy. Also, two familial cases were reported. The present systematic review highlights the necessity of having a high index of suspicion of PTS in patients with previous SARS-CoV-2 infection, as the clinical manifestations can be variable.

Nonetheless, a standardized approach is needed in order to investigate and report on PTS. Future studies should aim for a comprehensive assessment of patients. Factors such as the characteristics of the patients, evolution, and treatments should be consistently assessed across studies. Also, a thorough differential diagnosis should be employed.

## Figures and Tables

**Table 1:** Pain characteristics in PTS patients.

Study	Pain Severity	Onset Timing (Day/Night)	Location of Pain	Sleep	Mechanical Sensitivity	Time between Pain and Other PT Symptoms	Duration
Ahorukomeye 2022 [25]	Severe	N/R	Contralateral (right) shoulder	N/R	N/R	2 days	Resolved (at 12 weeks follow-up)
Alvarado 2021 [26]	Severe	N/R	Both shoulders	Disrupted sleep	N/R	N/R	N/R
Alvarez 2021 [27]	Unclear	N/R	Left shoulder	N/R	N/R	N/R	Resolved (at 3 months follow-up)
Ansari 2022 [28]	Severe	N/R	Cervical spine, left scapular region	N/R	N/R	N/R	Resolved (at 2 months follow-up)
Cabona 2021 [29]	Unclear	N/R	Left shoulder	N/R	N/R	N/R	Resolved (at 1-month follow-up)
Cabrera 2022 case 1 [30]	Severe	N/R	Left shoulder	N/R	N/R	N/R	Resolved within 26 days
Cabrera 2022 case 2 [30]	Severe	N/R	Left shoulder	N/R	N/R	N/R	Resolved within 21 days
Cacciavillani 2021 [31]	Severe	N/R	Left wrist and upper limb in the distribution of the lateral antebrachial cutaneous nerve	N/R	N/R	N/R	Resolved after 2 weeks
Castaneda 2022 case 1 [32]	Severe	N/R	Left shoulder	Sleep disruption	Exacerbated by movement	N/R	Occasional aching pain (at 4 months follow-up)
Castaneda 2022 case 2 [32]	Unclear	N/R	Left shoulder, radiating below elbow	Sleep disruption, worse at night	N/R	N/R	Improvement (at 9 months follow-up)
Castaneda 2022 case 3 [32]	Unclear	N/R	Left shoulder, radiating to the neck	N/R	N/R	A few weeks following onset of pain	N/R
Cheung 2022 [33]	Severe	N/R	Extended from the neck and right interscapular region to the shoulder and the ulnar side of the right arm and forearm	Sleep disruption	Aggravated by movement	N/R	Little or no pain (at 3 months follow-up)
Coll case 1 [34]	Unclear	N/R	Right shoulder	N/R	N/R	N/R	N/R
Coll case 2 [34]	Unclear	N/R	Left shoulder	N/R	N/R	N/R	N/R
Diaz 2021 [35]	Unclear	N/R	Right shoulder	N/R	N/R	7 weeks after pain onset	N/R
Fortanier 2022 case 1 [36]	Unclear	N/R	Right shoulder	N/R	N/R	Low interval	Resolution (at 3 months follow-up)
Fortanier 2022 case 2 [36]	Severe	N/R	Right shoulder	N/R	N/R	N/R	Pain persisted (4-month follow-up)
Ismail 2021 [37]	Severe	N/R	Left shoulder, followed by right shoulder one week later. Afterward, the pain intensified and progressed to both forearms and hands	N/R	Aggravated by touch and movement	2 weeks	Partial relief (8 weeks follow-up)
Mitry 2021 [38]	Severe	N/R	Multifocal joint pain, most prominent in the left shoulder and left hand. Followed by abdominal pain	N/R	Aggravated by movement	Shortly after onset	N/R
Ng 2022 [39]	Unclear	N/R	Left shoulder	N/R	N/R	N/R	Resolved (day 13)
Queler 2021 [40]	Severe	N/R	Left upper limb	N/R	N/R	N/R	N/R
Saade 2022 [41]	Unclear	N/R	Cervical spine and right upper limb	N/R	N/R	N/R	Resolved (6 months follow-up)
Siepmann 2020 [42]	Severe	N/R	Right shoulder, with a subsequent gradual shift to forearm and hand	N/R	Aggravated by arm extension	2 weeks	Partial relief (at 1.5 months after onset)
Viatgé 2021 [43]	Absent	N/A	N/A	N/A	N/A	N/A	N/A
Voss 2022 [44]	Severe	N/R	Left shoulder	Sleep disruption	Not alleviated or aggravated by shoulder movement	1 week	Important relief at 1 week
Zazzara 2022 [45]	Severe	N/R	Chest pain radiating to the proximal left arm, the shoulder and the upper region of the homolateral hemithorax	N/R	N/R	N/R	Persistence of painful dysesthesia (at 2 months from symptom onset). Improvement (at 4 months)

Notes: PTS: Parsonage-Turner syndrome; N/R: not reported; N/A not applicable.

**Table 2:** Clinical characteristics of PTS patients.

Study	Pain	Motor Deficit	Muscle Wasting	Paresthesia	Sensory Loss	Notes
Ahorukomeye 2022 [25]	Present	Absent	Present	Present	Absent	
Alvarado 2021 [26]	Present	Present	Present	Present	Present	Bilateral PTS
Alvarez 2021 [27]	Present	Present	Present	Present	Absent	Extended PTS
Ansari 2022 [28]	Present	Present	Absent	Absent	Present	
Cabona 2021 [29]	Present	Present	Present	Absent	Absent	Musculocutaneous nerve
Cabrera 2022 case 1 [30]	Present	Absent	Present	Present	Absent	Family case
Cabrera 2022 case 2 [30]	Present	Absent	Present	Present	Absent	Family case
Cacciavillani 2021 [31]	Present	Absent	Absent	Absent	Present	Pure sensory
Castaneda 2022 case 1 [32]	Present	Present	Present	Absent	Absent	
Castaneda 2022 case 2 [32]	Present	Present	Present	Present	Absent	
Castaneda 2022 case 3 [32]	Present	Present	Present	Present	Absent	
Cheung 2022 [33]	Present	Present	Absent	Present	Present	
Coll case 1 [34]	Present	Present	Present	Absent	Absent	Accessory nerve
Coll case 2 [34]	Present	Present	Present	Absent	Absent	Accessory nerve
Diaz 2021 [35]	Present	Present	Present	Absent	Absent	
Fortanier 2022 case 1 [36]	Present	Present	Absent	Absent	Absent	
Fortanier 2022 case 2 [36]	Present	Present	Absent	Absent	Absent	
Ismail 2021 [37]	Present	Present	Present	Present	Present	Bilateral PTS
Mitry 2021 [38]	Present	Present	Absent	Absent	Absent	Diaphragm
Ng 2022 [39]	Present	Present	Absent	Present	Absent	
Queler 2021 [40]	Present	Present	Absent	Present	Absent	
Saade 2022 [41]	Present	Present	Present	Absent	Absent	
Siepmann 2020 [42]	Present	Present	Absent	Present	Present	
Viatgé 2021 [43]	Absent	Present	Absent	Absent	Present	
Voss 2022 [44]	Present	Present	Absent	Absent	Present	
Zazzara 2022 [45]	Present	Absent	Absent	Absent	Present	Pure sensory

**Table 3:** Characteristics of patients with PTS that necessitated ICU treatment.

Study	ICU Stay	Prone Positioning	PTS Symptoms
Alvarado 2021 [26]	Several days	On 2 occasions	During a visit to the rehabilitation department
Alvarez 2021 [27]	23 days	Intermittently	8 days after extubation
Cabona 2021 [29]	13 days	N/R	At ICU discharge
Castaneda 2022, case 2 [32]	Several weeks	N/R	Following extubation
Coll 2021, case 1 [34]	6 weeks	Present	1 month after ICU discharge
Coll 2021, case 2 [34]	5 weeks	Present	1 week after ICU discharge
Queler 2021 [40]	7 weeks	None	Following extubation
Viatgé 2021 [43]	24 days	Present	Following extubation

Notes: ICU—intensive care unit; PTS—Parsonage-Turner syndrome; N/R—not reported.

## Data Availability

All data for the systematic review are available within the article.

## Supplementary Materials

The following supporting information can be downloaded at: https://www.mdpi.com/article/10.3390/biomedicines11030837/s1, Supplemental Materials S1: PRISMA checklist [64]; Supplemental Materials S2: Characteristics of the included studies.
